# Positive and negative ageing perceptions account for health differences between older immigrant and native populations in the Netherlands

**DOI:** 10.1186/s12877-021-02119-8

**Published:** 2021-03-18

**Authors:** Anna P. Nieboer, Thijs van den Broek, Jane M. Cramm

**Affiliations:** Department of Socio-Medical Sciences, Erasmus School of Health Policy & Management, Erasmus University Rotterdam, Burgemeester Oudlaan 50, 3000 DR Rotterdam, the Netherlands

**Keywords:** Ageing perception, Self-rated health, Immigrant, Native, Netherlands

## Abstract

**Background:**

This study assessed the extent to which persistent differences in self-rated health (SRH) between older immigrants and natives are attributable to negative and positive ageing perceptions.

**Methods:**

The study was conducted with three population groups in Rotterdam, the Netherlands: native Dutch people aged ≥70 years (*n* = 1150), Turkish immigrants aged ≥65 years (*n* = 680) and Moroccan immigrants aged ≥65 years (*n* = 292). To assess participants’ internal ageing representations, we used the short Aging Perceptions Questionnaire, which distinguishes negative (consequences, chronic and cyclical timeline awareness, and emotional representations) and positive (positive consequences, positive and negative control) dimensions and has been validated in native and immigrant populations. We analysed differences in ageing perceptions between immigrants and natives and the associations of ageing perceptions with SRH. We used Karlson–Holm–Breen decomposition to assess ageing perceptions’ mediation of the relationship between migration background and SRH.

**Results:**

Older immigrants had stronger negative and weaker positive ageing perceptions (excepting the positive consequences of ageing) than did Dutch natives. Ageing perceptions mediated the relationship between migration background and SRH. SRH differences between Turkish immigrants and native Dutch older persons were explained mainly by differences in negative consequences and cyclical timeline awareness. SRH differences between Moroccan immigrants and native Dutch older persons were attributable mainly to differences in negative consequences and positive control.

**Conclusions:**

Differences in positive and negative ageing perceptions between older immigrants and natives in the Netherlands largely explained SRH differences between these population groups.

## Background

In the Netherlands, most immigrants live in large cities and Turks and Moroccans comprise the largest non-western immigrant groups [1]. Rapid population ageing among these immigrant groups has been noted and is expected to continue in the next decades [2]. Older migrants report poor health, functional limitations and chronic diseases, and make more use of health care than do older natives, in the Netherlands [3–6] and in Europe overall [7–9]. These persistent health differences can be attributed only partly to differences in socioeconomic status, and research has highlighted the potential importance of ageing perceptions [10–13].

Ageing perceptions have been shown to be important predictors of health outcomes and mortality among older people [14–16]. According to Barker et al. [17], ageing perceptions are multidimensional, as people build complex schemas to make sense of the multifaceted ageing process. These dimensions of ageing perceptions can be positive (e.g. related to ongoing personal growth and development, leading to better outcomes [15, 16]) and negative (e.g. related to coping with declines [18]). Inspired by Leventhal et al.’s [19, 20] self-regulation model (SRM), Barker and colleagues [17] identified ageing perceptions in the following dimensions: i) the timeline, referring to a person’s awareness and longitudinal experience of ageing, which can be *chronic* (constant), *acute* or *cyclical* (varying over time, e.g. ‘I go through phases of feeling old’); ii) consequences, referring to the believed impact of ageing on various life domains, which can be *positive* (e.g. ‘as I get older, I get wiser’) or *negative* (e.g. ‘as I get older, I can take part in fewer activities’); iii) control, referring to beliefs about personal ways of managing one’s experience of ageing, which can be *positive* (e.g. ‘whether I continue living life to the full depends on me’) or *negative* (e.g. ‘slowing down with age is not something that I can control’); and iv) emotional representations, referring to negative emotional reactions to ageing (e.g. ‘I get depressed when I think about getting older’).

Ageing perceptions are known to differ between native and immigrant older populations [21, 22]. In general, older immigrants in the Netherlands experience ageing more negatively than do Dutch natives, which is partly attributable to financial problems, distrust in the Dutch social system, language barriers and unfulfilled care expectations [23–25]. Turkish and Moroccan immigrants tend to have relatively low degrees of perceived *control* [26, 27], which may correspond to more defeatist expectations of old age, in turn leading to more negative experiences of perceived personal control of ageing management. Turkish people are known to consider ‘old age’ to begin at a much earlier stage than do people originating from western countries, which is expected to lead to more negative *timeline* perceptions (e.g. [28]). Given their health impact, ageing perceptions thus can be expected to contribute to health differences between Turkish and Moroccan immigrants and Dutch natives with no migration background. The extent to which ageing perceptions differ among older Turkish, Moroccan and Dutch people, and the manner in which any such difference contributes to persistent health differences, however, remain unclear. Thus, this study was conducted to assess the extent to which persistent differences in self-rated health (SRH) between these immigrant and native populations in the Netherlands are attributable to differences in negative and positive ageing perceptions.

## Methods

This study was conducted with three population groups in Rotterdam, the Netherlands: Dutch natives aged ≥70 years (*n* = 1150), Turkish immigrants aged ≥65 years (*n* = 680) and Moroccan immigrants aged ≥65 years (*n* = 292).

### Older Dutch natives with no migration background

The first dataset was obtained with a sample of 2890 independently living older adults selected from the Rotterdam population register in 2013. Sampling was random and stratified by age group (70–74, 75–79, 80–84 and ≥ 85 years) and neighbourhood. The number of participants per neighbourhood was weighted proportionally to the district population ratio. Eligible older adults were mailed a questionnaire to fill in manually, with an invitation to participate in the study and a pre-addressed envelope for questionnaire return. Two reminders were sent in cases of non-response. Sixty-seven older adults who resided in nursing homes or were hospitalised, and 25 older adults who could not participate due to serious medical issues (i.e. dementia) or death, were excluded before the study began. Of the remaining 2798 respondents, 1280 participated in the survey (46% response rate). One hundred thirty of these respondents were excluded because they were immigrants, leaving a total of 1150 native Dutch respondents.

### Older Turkish immigrants

The second dataset comprised data from 680 older Turkish immigrants, gathered between March 2015 and February 2016. Individuals (*n* = 2350) were sampled randomly from the Rotterdam municipal register; 213 of these individuals were ineligible due to serious medical issues, death, change of address or non-Turkish ethnic background, leaving a total of 2137 eligible older Turkish immigrants. Each of these individuals was mailed a questionnaire (in Dutch and Turkish) with an invitation to participate in the study and a pre-addressed envelope for questionnaire return. In cases of non-response, a reminder was sent by mail, followed by a face-to-face interview in the individual’s home. This strategy resulted in a 32% response rate (*n* = 680).

### Older Moroccan immigrants

The third dataset comprised data from 292 older Moroccan immigrants, gathered in 2017 and the beginning of 2018. Individuals (*n* = 1491) were sampled randomly from the Rotterdam municipal register; 77 of these individuals were ineligible due to serious medical issues, death, change of address or admission to a hospital or long-term care facility, leaving a total of 1414 eligible older Moroccan immigrants. Each of these individuals was mailed a questionnaire (in Dutch, Berber and Arabic) with an invitation to participate in the study and a pre-addressed envelope for questionnaire return. In cases of non-response, a reminder was sent, followed by face-to-face interviews at people’s homes, resulting in a 21% response rate (*n* = 292).

### Ethical approval

According to the Central Committee on Research Involving Human Subjects (CCMO), the current study did not fall within the scope of the Medical Research Involving Human Subjects Act and thus did not require prior review by an accredited medical research and ethics committee or the CCMO. All respondents were informed about the aims of the study and assured that participation was anonymous and voluntary prior to providing consent to participation.

### Measures

We used the short (21-item) Aging Perceptions Questionnaire (APQ-S), which has been validated in native and immigrant populations [29, 30], to assess participants’ ageing perceptions. The APQ-S assesses seven dimensions of ageing perceptions identified by Barker and colleagues [17] based on the SRM [19]: chronic and cyclical awareness of the ageing timeline, positive and negative experiences with the consequences of ageing, positive and negative feelings about one’s control of the ageing process, and negative emotional reactions to ageing. Each dimension is assessed using three items, with responses ranging from 1 (‘totally disagree’) to 5 (‘totally agree’). Items for the negative control dimension were reverse coded so that higher scores indicated more perceived control [13, 22, 29, 30].

SRH was assessed by asking respondents to rate their perceived general health on a five-point scale (ranging from 1 [‘poor’] to 5 [‘excellent’]), which is known to be a good indicator of general health and a strong predictor of mortality [31, 32]. For simplicity and ease of interpretation, and following previous studies [33, 34] the outcome variable was dichotomised into responses ‘good’ to ‘excellent’ (0) and ‘less than good’ (1).

The questionnaire also solicited data on respondents’ age, gender, highest educational level (in the Netherlands or abroad), monthly household income (including social benefits, pensions and alimony) and marital status. Educational level was dichotomised as low (1; completion of primary education or less) and not low (0; more than primary education). Income level responses (ranging from 1 [‘less than €1,000 a month’] to 4 [‘€3,050 or more a month’]) were dichotomised as low (1; less than €1350 a month) and not low (0; €1350 or more a month). Marital status (married, divorced, widowed, single or cohabitating) was dichotomised as unpartnered (1; divorced, single or widowed) and partnered (0; married or cohabitating).

### Statistical analysis

*F* tests were performed to assess whether ageing perceptions differed systematically among study groups with posthoc pairwise comparisons (Tukey test) to determine exactly which means differed significantly. We then estimated logistic regression models to predict the odds of having less than good SRH. In the first model, these odds were regressed on migration status and background characteristics. Ageing perception data (by dimension) were added to the second model. We performed a formal mediation analysis using the Karlson–Holm–Breen (KHB) decomposition method [35] to assess the extent to which group differences in SRH were attributable to differences on the seven ageing perceptions. The KHB method was developed specifically for the analysis of mediation in logistic regression and other nonlinear models, and it accounts for the attenuation bias that may occur in such models.

#### Missing values

Information on at least one variable of interest was missing for 506 (23.9%) respondents. The variables with the most missing values were income (*n* = 340), *cyclical timeline awareness* (*n* = 102) and *emotional representations of ageing* (*n* = 102). Multiple imputation with chained equations was used to deal with missing information. The underlying missing at random assumption holds that any difference in distribution between missing and observed values can be explained by variables included in the imputation model [36]. We estimated separate imputation models for each of the three study groups. The results of substantive analyses of 20 imputed datasets were combined into a single set of results following Rubin’s rules [37].

## Results

Table 1 displays descriptive statistics for the study samples. Less than good self-rated health was reported by 44.7% of native Dutch, 72.6% of Turkish immigrants and 67.3% of Moroccan immigrants. Mean age ranged from 72.9 for Turkish immigrants to 78.9 for native Dutch.

**Table 1:** Characteristics of Native Dutch and Turkish and Moroccan Immigrants

	Native Dutch (*n* = 1150)	Turkish origin (*n* = 680)	Moroccan origin (*n* = 292)
Less than good self-rated health	44.7%	72.6%	67.3%
Female	58.3%	47.6%	42.8%
Mean age, years (standard deviation)	78.9 (6.3)	72.9 (5.0)	73.8 (6.1)
Unpartnered	58.9%	28.6%	24.9%
Low education	15.6%	80.2%	86.5%
Low income	35.3%	84.2%	78.4%

Multiple imputation using chained equations was used to deal with missing values

More than half of the native Dutch respondents (58.3%) were female versus 47.6% of Turkish immigrants and 42.8% of Moroccan immigrants. Large differences were found in being unpartnered, with native Dutch respondents being unpartnered more often than Turkish and Moroccan immigrants. As for education and income, Turkish and Moroccan immigrants more often reported low income and education levels as compared to their native Dutch counterparts. These variables were controlled for in the multivariate analyses.

Ageing perceptions in all seven dimensions differed systematically among the study groups. Turkish and Moroccan immigrants had significantly stronger negative (*consequences*, *chronic* and *cyclical timeline awareness*) and weaker positive (*negative* and *positive control*) ageing perceptions than did native Dutch respondents. Turkish immigrants had stronger *emotional representations* than did native Dutch respondents (meaning that they had more negative, depressive emotional responses to ageing), whereas Moroccan immigrants reported weaker *emotional representations* than did Turkish and native respondents. Turkish immigrants reported stronger positive experiences with the *consequences* of ageing than did native Dutch and Moroccan respondents (Table 2).

**Table 2:** Ageing Perceptions

	Native Dutch (*n* = 1150)	Turkish origin (*n* = 680)	Moroccan origin (*n* = 292)	Group differences
Positive ageing perceptions				
Consequence positive	3.38 (0.76)^a^	3.57 (0.98)^ac^	3.39 (0.77)^c^	*F*(2, 2119) = 11.3, *p* < .001
Control positive	3.72 (0.69)^ab^	3.44 (0.98)^ac^	3.22 (0.87)^bc^	*F*(2, 2119) = 52.8, *p* < .001
Control negative	2.42 (0.77)^ab^	2.25 (0.86)^a^	2.28 (0.74)^b^	*F*(2, 2119) = 9.6, *p* < .001
Negative ageing perceptions				
Consequence negative	3.39 (0.83)^ab^	3.84 (0.94)^a^	3.74 (0.88)^b^	*F*(2, 2119) = 58.3, *p* < .001
Timeline chronical	3.34 (0.92)^ab^	3.77 (0.92)^a^	3.71 (0.80)^b^	*F*(2, 2119) = 53.5, *p* < .001
Timeline cyclical	2.84 (0.88)^ab^	3.52 (0.75)^ac^	3.14 (0.80)^bc^	*F*(2, 2119) = 140.6, *p* < .001
Emotional representations	2.45 (0.85)^ab^	2.87 (1.03)^ac^	2.30 (0.90)^bc^	*F*(2, 2119) = 55.6, *p* < .001

Data are presented as mean (standard deviation)

^abc^ Posthoc pairwise comparisons to determine which means differ significantly (*p* < 0.05)

Multiple imputation using chained equations was used to deal with missing values

**Fig. 1:**
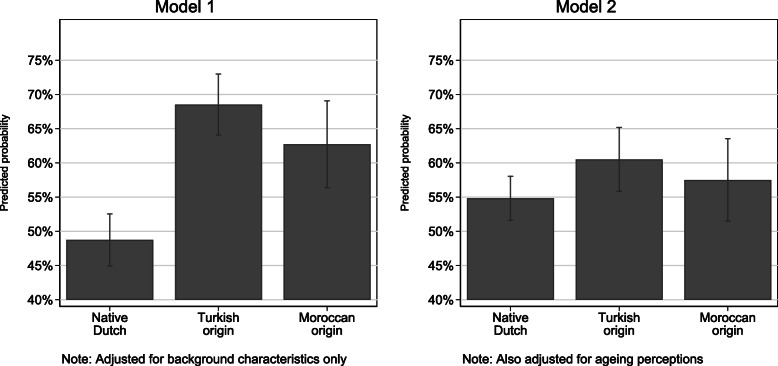
Adjusted Predictions of the Probability of Less than Good Self-Rated Health by Group

The first logistic regression model (adjusted for gender, marital status, educational level, income and age) showed that immigrants of Turkish and Moroccan origins were more likely to report less than good SRH than were their native Dutch counterparts. It also showed that female gender, low educational level, low income and older age were associated with a greater likelihood of reporting less than good SRH (Table 3). In the second model (adjusted for migration background and background characteristics), positive *consequences* and *control* perceptions of ageing were associated significantly with a lower likelihood of reporting less than good SRH. Negative *consequences* and *cyclical timeline* perceptions were associated significantly with a greater likelihood of reporting less than good SRH (Table 3). With the addition of ageing perceptions to the model, the coefficient estimates for immigrant versus native origin were substantially smaller than in the first model, and no longer significant. Adjusted predictions of the likelihood of reporting less than good SRH are presented in Fig. 1 to facilitate a more intuitive interpretation of the magnitude of the health differences among groups before and after adjustment for ageing perceptions. The adjusted predictions were calculated by setting the migration status to one group at a time and using observed values for each case for all other covariates included in each model. The predicted probability of less than good SRH was then derived for each case based on these observed values, and a mean predicted probability value was calculated. Compared with the native Dutch group, people of Turkish and Moroccan origin had, respectively, 20 percentage point (95% confidence interval: 0.129, 0.267; *p* < 0.001) and 14 percentage point (95% confidence interval: 0.056, 0.223; *p* < 0.001) greater predicted probabilities of less than good SRH before adjustment for ageing perceptions. After adjustment for ageing perceptions (Fig. 1, model 2) the differences in the predicted probability of less than good SRH with the native Dutch group were considerably smaller and no longer significant for the Turkish origin (6 percentage points; 95% confidence interval: − 0.010, 0.124; *p* = 0.097) and Moroccan origin (3 percentage points; 95% confidence interval: − 0.050, 0.104; *p* = 0.495) groups.

**Table 3:** Results of Logistic Regression Models of Less than Good Self-Rated Health (*n* = 2122)

	Model 1			Model 2		
	Coefficient	(SE)	Odds ratio	Coefficient	(SE)	Odds ratio
Migration background						
Native Dutch	Ref.			Ref.		
Turkish origin	0.86***	(0.15)	2.37	0.31	(0.18)	1.36
Moroccan origin	0.59**	(0.18)	1.81	0.14	(0.21)	1.15
Background characteristics						
Female	0.41***	(0.10)	1.50	0.45***	(0.12)	1.56
Unpartnered	−0.11	(0.12)	0.90	−0.11	(0.13)	0.89
Low education	0.46***	(0.13)	1.59	0.30*	(0.15)	1.35
Low income	0.39**	(0.12)	1.47	0.29*	(0.14)	1.34
Age	0.02*	(0.01)	1.02	−0.02*	(0.01)	0.98
Positive ageing perceptions						
Consequence positive				−0.23**	(0.07)	0.80
Control positive				−0.38***	(0.08)	0.68
Control negative				−0.08	(0.08)	0.92
Negative ageing perceptions						
Consequence negative				0.86***	(0.08)	2.36
Timeline chronical				0.10	(0.07)	1.11
Timeline cyclical				0.30***	(0.08)	1.35
Emotional representations				0.09	(0.07)	1.09
Constant	−2.11***	(0.64)		−0.86	(0.87)	
Pseudo *R*^2^	.08			.21		

Multiple imputation using chained equations was used to deal with missing values

*p < 0.05, **p < 0.01, ***p < 0.001

Ageing perceptions significantly mediated portions of the health differences between the Turkish and Moroccan immigrant groups and the native Dutch group. These perceptions explained 72% of the difference in SRH between Dutch and Turkish respondents, after adjustment for gender, marital status, educational level, income and age. This percentage was 81% for the difference in SRH between Dutch and Moroccan respondents (Table 4).

**Table 4:** Decomposition of Perception Coefficients According to Migration Background

	Turkish origin vs native Dutch					Moroccan origin vs native Dutch				
	B	(SE)	*p*	Share total effect (%)	Share indirect effect (%)	B	(SE)	*p*	Share total effect (%)	Share indirect effect (%)
Reduced-form model	1.08	(0.18)	<.001	100.0		0.74	(0.20)	<.001	100.0	
Full model	0.31	(0.21)	.092	28.3		0.14	(0.21)	.494	19.4	
Δ Reduced-form model - Full model	0.78	(0.13)	<.001	71.7	100.0	0.59	(0.13)	<.001	80.6	100.0
Components of difference										
Positive ageing perceptions										
Consequence positive	−0.06	(0.02)	.017	−5.2	−7.3	−0.02	(0.02)	.261	−2.1	−2.7
Control positive	0.10	(0.03)	.001	9.7	13.5	0.19	(0.05)	<.001	25.3	31.3
Control negative	0.02	(0.02)	.250	1.4	1.9	0.01	(0.01)	.263	1.6	1.9
Negative ageing perceptions										
Consequence negative	0.41	(0.06)	<.001	37.7	52.6	0.29	(0.07)	<.001	39.3	48.8
Timeline chronical	0.05	(0.03)	.137	4.3	6.0	0.04	(0.03)	.146	4.8	6.0
Timeline cyclical	0.23	(0.06)	.001	20.8	29.0	0.11	(0.04)	.005	14.3	17.8
Emotional representations	0.03	(0.03)	.193	3.1	4.4	−0.02	(0.02)	.215	−2.5	−3.1

SRH differences between the Turkish origin and native Dutch groups were explained mainly by differences in *negative consequences* (53% of the indirect effect) and *cyclical timeline awareness* (29% of the indirect effect). SRH differences between the Moroccan origin and native Dutch groups were attributable mainly to differences in *negative consequences* (49% of the indirect effect) and *positive control* (31% of the indirect effect). Furthermore, the KHB mediation analysis revealed a significant suppression effect for *positive consequences* for the Turkish origin group, indicating that the health difference relative to the native Dutch group was larger, rather than smaller, after adjustment for this ageing perception dimension. This result is not surprising, given the high *positive consequences* scores in the Turkish origin group (Table 2).

## Discussion

This study suggests that ageing perceptions may play crucial roles in persistent health differences between older immigrants and natives. We found that older immigrants of Turkish and Moroccan origin generally had stronger negative and weaker positive ageing perceptions than did native Dutch persons without a migration background. One exception was that the Moroccan group had more favourable *emotional representations* scores, indicating weaker negative emotional responses to the thought of ageing, than did the native Dutch group. A second exception was that the Turkish group had relatively favourable *positive consequences* scores, indicating a tendency to have relatively positive expectations about the impacts of ageing on various life domains. In previous studies, older immigrants have reported recognition of a few positive aspects of ageing, such as having more free time and moments of rest and opportunities to establish enjoyable social relationships [24, 25]. In Muslim cultures, family interdependencies are moreover stronger; people spend more time with older family members, and older people are the most valued and respected in the social hierarchy [38, 39]. These factors may explain Turkish immigrants’ higher *positive consequences* scores relative to those of native Dutch respondents, but they do not explain Moroccan immigrants also have significantly lower scores than their Turkish counterparts. The lower *positive* and *negative control* scores among immigrants than among native Dutch people may be explained by older immigrants’ concept that what happens during ageing, including whether one becomes ill, may be in God’s hands alone (‘*inshallah*’ [40];). These findings are in line with those of Mayer and colleagues [39], who found that autonomy and/or control are typically valued less in Turkey than in western countries. Overall, these findings support the notion – based on previous qualitative findings – that perceptions of ageing are multidimensional, and that older immigrants in the Netherlands generally have more negative ageing perceptions compared with natives, but still can perceive relative success with a specific aspect of ageing [41].

The results of our analyses also suggested that differences in ageing perceptions between immigrant and native groups contributed markedly to group differences in health. After adjustment for ageing perceptions, the group differences in health were small and not significant. Differences in SRH from that of native Dutch older people were explained mainly by differences in *negative consequences* (Turkish and Moroccan immigrants), *cyclical timeline awareness* (Turkish immigrants) and *positive control* (Moroccan immigrants) scores, suggesting that pessimistic ideas about the impact of ageing on various life domains, cyclical awareness of ageing and low confidence in the management of different aspects of ageing negatively shape the health of older immigrants. It also underlines the importance of a positive outlook on health (e.g. [40]). These findings highlight the potential influence of positive and negative ageing perceptions on health among immigrant populations. They suggest that health outcomes among these vulnerable groups may be improved through interventions beyond the current scope of the health system. Interventions aimed at having a positive outlook on health in later life, at highlighting the positive consequences of ageing and at working on confidence among immigrants in the management of different aspects of ageing could be particularly promising.

Several limitations of this study should be taken into account when interpreting our findings. First, the cross-sectional design prevented us from drawing conclusions about causality. The relationships between ageing perceptions and health are probably partly bi-directional. Given that Wurm and colleagues [42] found that ageing-related cognitions had greater impacts on changes in health than vice versa, we expect the strongest direction of influence to be from ageing perceptions to health. Second, we examined ageing perceptions among only three groups of older people in the Netherlands; more studies in other countries and among other immigrant groups are needed to increase our understanding of ageing perceptions across groups according to country of origin, and the roles of ageing perceptions in older peoples’ health. Third, the relatively low response rates, especially among Moroccan immigrants, may have influenced our study findings. Older adults in poorer health may not have filled in the questionnaire. Thus, the actual number of people with less than good SRH may be larger than detected in this study. Finally, the age of the respondents differs between native and immigrant older adults. We included natives in the ages of 70 years or older and immigrants aged 65 years or older. While old age is considered to begin at an earlier stage according to Turkish people than people from western cultures ([e.g. [28]), this still affects comparison between groups. The results of the analyses were similar when we only included immigrants aged 70 years or older as well. As we controlled for age in the multivariate analyses, we decided to keep 65 as a threshold for the immigrant groups.

## Conclusions

Differences in positive and negative ageing perceptions between older immigrants and natives in the Netherlands largely explain the persistent differences in SRH between these populations. These findings should be taken as a call to action to change ageing perceptions among immigrants early in their lives, which may be expected to benefit their health in later life.

## Data Availability

The datasets analyzed during the current study are available from the corresponding author on reasonable request.

## Abbreviations

APQ-S: Short (21-item) Aging Perceptions Questionnaire
CCMO: Central Committee on Research Involving Human Subjects
KHB: Karlson–Holm–Breen
SRH: Self-rated health
SRM: Self-regulation model
